# Exploring resilience in sports participation and self-assessed leadership among women in Malaysia: a mixed-methods approach

**DOI:** 10.3389/fspor.2026.1890468

**Published:** 2026-07-06

**Authors:** Jia Wen Foo, Agnes Kye Lynn Tam, Dayang Adlina Sharine Abang Abdillah, Syarifah Fathynah, Po Ling Chen

**Affiliations:** 1 School of Psychology, Faculty of Science and Engineering, University of Nottingham Malaysia, Semenyih, Selangor, Malaysia; 2 National Sports Institute of Malaysia, Kuala Lumpur, Malaysia

**Keywords:** gender issues, Malaysia, resilience, sports gender equality, women in sports

## Abstract

**Introduction:**

Women's sports participation in Malaysia remains disproportionately low despite increasing national emphasis on sports gender equity. Limited empirical literature examining the subject using an integrated sociocultural and psychological perspective in the Malaysian cultural context. This study aimed to assess women resilience in sports in terms of self-perceived efficacy, emotions, self-rated problem-solving, self-assessed leadership and barriers to continuous sports participation.

**Methods:**

A mixed-methods approach which consists of an online survey and focus groups interviews targeted Malaysian women was employed. Quantitative data were collected from 191 participants using the Resilience Scale, Leadership/Teamwork Self-Efficacy Scale, Positive and Negative Affect Schedule, Shortened Problem-Solving Skills and Leadership Self-Assessment, whilst qualitative data were collected from two separate focus groups comprising women who were active (*n* = 6), and inactive (*n* = 7) in sports.

**Results:**

Survey data analysis demonstrated Leadership and Teamwork Self-Efficacy, Positive Affect and Problem-Solving Skills significantly accounted for variance in Resilience. Thematic analysis revealed five key themes: (1) barriers to sustained sports participation, (2) gender influences and societal expectations, (3) community, motivation, and leadership, (4) the role of social media in empowerment, and (5) policy awareness and institutional support.

**Discussion:**

These findings provide insights for future longitudinal or interventions studies targeting factors account for variance in building resilience as an internal resource for women in sports, while highlighting systemic and sociocultural barriers hindering women's sports participation that should be addressed to further gender equity in the Malaysian sports environment.

## 1 Introduction

Over the past few decades, women's participation in sports worldwide has soared to record highs (1). In Malaysia, female athletes have demonstrated what women's athleticism can achieve in sports. From diver Pandelela making the mark as the nation's first female athlete to nab an Olympic bronze medal in 2012, to now-retired world renowned world squash champion Nicol Ann David nurturing young athletes via her non-profit Nicol David Organisation with co-founder Mariana de Reyes, these sportswomen are role models and inspiration in the gender equality movement, reinforcing women's equal right to hold space in sports. However, a shift in focus to the broader Malaysia population shows a different side of the story. Although the Malaysian Sports Culture Index scores improved from 2021 to 2023 (2–5), indicating a general increase in sports involvement, women's participation has consistently lagged behind their male counterparts, remaining at an overall low participation (46.1 compared to 62.9 respectively).

Despite these participation gains and global efforts, women remain significantly underrepresented in leadership positions across sporting institutions (6, 7). Persistent barriers—including gender stereotypes, lack of support, limited access to networks, and structural inequalities—hinder their advancement into decision-making roles (8–10). While much of this research originates from Western contexts, less is known about the lived experiences of women in sports leadership in Southeast Asia, particularly in Malaysia. In this context, cultural norms, societal expectations, and limited institutional support uniquely shape women's participation and progression (6). Although national policies aim to increase female representation, anecdotal and media reports suggest that women continue to face challenges beyond policy provisions, indicating a gap between intention and lived experience (11).

### 1.1 Resilience as an internal resource

Resilience refers to an individual's capacity to adapt positively to adverse situations, thereby maintaining physical and psychological well-being in the face of stress and trauma (12). It is a dynamic process that can be improved on through interventions rather than a static trait that remains unchanged over time, marking it a favourable central construct in intervention development (13). Research has linked resilience to favourable outcomes including stronger academic engagement and performance (14), as well as reduced interruptions at a task despite challenges (15). In sports, higher levels of resilience are linked to better performance, as well as reduced psychological stress among athletes (16). In women's sports, resilience can be a valuable skill in facilitating their continued participation, particularly in supporting them in problems such as gender inequality, body image and burnout (17), especially considering the moderating role of resilience of harmful effects from prejudiced environments (18).

Hence, the promising potential of resilience as an internal, personal resource that women can readily access and build upon, particularly in its potential against the complex and challenging gendered sports environment could be further explored. In particular, furthering the understanding of factors that are accountable to building resilience could provide directions to designing actionable resilience interventions.

The supporting factors to resilience encompass external (e.g., having emotional responsive relationships, inclusive prosocial environments) and internal sources (e.g., emotional regulation skills, mindfulness) (13, 18–20). This study examines the relationship of emotions, self-efficacy and problem-solving skills with resilience to explore its role as a readily accessible internal resource to empower women in sports. Adapting the theoretical framework developed by Pillay-Naidoo and Nel (18), the consideration of cognitive (self-efficacy), emotional (positive and negative emotions) and behavioural (problem solving) domains to resilience is of interest in this study.

### 1.2 Leadership and teamwork self-efficacy

Self-efficacy is defined as an individual's belief in their ability to accomplish a given task, influencing behaviour, motivation and how one responds to difficult tasks (21). Individuals with high self-efficacy are more likely to attempt, put more effort, persist and achieve better results than those with low self-efficacy (22). This is because individuals with high self-efficacy tend to view difficulties as challenges instead of threats, which reduces negative emotions, whether to themselves or the situation, when facing adversity (23). This fosters a sense of control over situations, allowing the belief that change is possible and they are able to influence the outcomes through their actions and in turn, empowers them to reject negative, unconstructive thoughts about themselves (24). In this study, the leadership and teamwork self-efficacy (LTSE) described by Chemers et al. (21) is examined. This is due to the importance of leadership traits in sports, as the presence of athlete leadership, whether formal or informal, improves overall performance and motivation in a sports team (25).

### 1.3 Positive and negative emotions

Positive Affects (PA) refers to pleasurable emotional experiences such as excitement and enthusiasm, whereas Negative Affects (NA) encompasses unpleasant emotions such as lethargy and anger (26). Growing research within the broaden-and-build theory underscores the role of PA in facilitating exploratory behaviours via contributing to one's ability to hold broader perspectives when evaluating problems, thereby enhancing adaptability, creativity and problem-solving abilities (27). This in turn improves the likelihood of discovering opportunities within stressful situations, increasing the probability of adaptive responses and improving task success. Over time, the compounding of positive emotions allows one to “build” psychological resources such as social support and emotional well-being (18), which in turn becomes supportive resources in future challenges.

Based on Thompson et al. (28), PA has shown to enhance performance and ability to bounce back from an unpleasant event in athletes. The ability of PA to broaden athletes' awareness supports their ability to respond with novel and creative solutions. This can translate to increased performance in sports competition and in turn, reports of increased pleasant experience in sports, higher resilience and task-oriented strategies. Conversely, NA narrows a person's thought action repertoire and is linked to increased stress due to a perception of lack of ability to affect changes when facing challenges (29). It is linked negatively with resilience as NA experiences tend to promote less effective coping strategies such as disengaging or distraction (28), and can even lead to dropping out of sports entirely (30).

### 1.4 Problem-solving skills

Problem solving refers to an individual's ability to cope and resolve challenges that arise (31). Ineffective problem solving is linked to maladaptive psychological outcomes such as increased stress, anxiety and procrastination (32). In contrast, adaptive problem-solving strategies are generally task-oriented, proactive, and involve deliberate planned actions to bring on favourable changes (e.g., solutions). As such, athletes with higher self-perceived problem-solving skills are more equipped to utilise thoughtful strategies under pressure (33), and tend to be more motivated and resilient as they are able to cope with difficulties and persevere (34).

### 1.5 Self-assessment as an accessible development tool

Self-assessment leadership development is a type tool that enables individuals to self-evaluate their leadership skills and behaviours, and supports self-empowered growth (35). Within the sports environment, where female sports leaders may receive less leadership opportunities due to issues of limited access to networking, mentorship and professional development (36), these tools can fill in the gap as an accessible, flexible development opportunity for women's leadership development. Studies have shown that women with higher self-confidence are more likely to perceive themselves as capable leaders, which enhances their problem-solving abilities and resilience (37). Moreover, perceived self-efficacy impacts women willingness to take on leadership roles in sports. For instance, female athletes who believe in their leadership abilities are more likely to pursue coaching and administrative positions (38). This self-belief not only helps them overcome barriers but also fosters personal strength and resilience, enabling them to navigate the challenges associated with sports leadership (37).

Advancing gender equity in Malaysian sports requires a comprehensive understanding of the underlying root causes that shape participation and leadership. Emphasis should be focused on transforming the sports landscape as a whole into an inclusive, equitable environment, rather than putting the onus of change completely on the shoulders of women. As presented by Pillay-Naidoo and Nel (18), external causes are the more significant threat against meaningful advancement, and remain a major instrument in perpetuating the cycle of inequality. However, changes take time, as it involves research, design and the implementation of appropriate measures. As such, exploring accessible internal resources that can be utilised by women currently navigating this challenging environment is of interest.

### 1.6 Barriers associated with the sports gender gap

The barriers of women's limited sports participation consist of multifaceted and intertwined factors (39), and can be categorised into three levels: (1) socio-cultural, (2) internal factors and (3) organisational and structural factors.

#### 1.6.1 Socio-cultural factors to women's sports participation

Historically, sports as a social institute have been male-dominated, and the male sports hegemony is propagated via the valuation of specific masculine-attributed traits in sports such as competitiveness, assertiveness and being in control (35, 36). Normative cultural perceptions that assign nurturing and caregiving roles to women reinforce a stereotypical narrative in which sports are predominantly viewed as a masculine domain within the broader socio-cultural context (40, 41), and the female gender is perceived to be incompatible and less suitable with sports involvement.

Consequently, female participation is often viewed as nonconformity to accepted gender roles, a perception that manifests in discriminatory behaviours such as teachers assuming girls lack interest in physical education or parents discouraging daughters from sports they deem inappropriate (42, 43). Girls themselves may avoid sports, especially those that are strongly masculine coded such as soccer, due to fearing being viewed as masculine and the consequential negative feedback and social exclusion from peers from such perceptions (44). Body image concerns further exacerbate this issue due to the erroneous association of sports with a muscular image (45). The multidirectional social pressure to conform to own-gender appropriate behaviour can thus push girls and women away from sports, with long lasting consequences on lifelong sports participation.

#### 1.6.2 Internal factors to motivation, self-perception and competing priorities

The internalisation of the females gender-role stereotypes and the perception of sports as a gendered activity occurs through socialisation, where societal norms and expectations shape individual's belief and behaviours (46). This is a result from repeated exposure of cultural expectations through interaction from social surroundings, especially via role modelling of parents and peers (e.g., social learning theory; 23). Although these biases tend to go unnoticed by an individual (47), they can have a very real impact in behaviour and decision-making, which can show up as a lack of interest and disengagement of physical education classes in school (48).

This process can be explained by the expectancy-value model, which posits that motivation is shaped by one's expectation of success in a task and the subjective value one assigns to it-value that can be intrinsic, functional, or related to satisfaction vs. perceived cost (49). These factors ultimately influence the likelihood of engaging in the behaviour. In sports, athletes with higher perceived self-competence and motivation reported higher intention to keep with competitive sports activities (48). However, societal stereotypes suggesting that girls are naturally less physically capable and thus unsuited for sports can undermine self-perceived athletic competence, which in turn diminishes the personal value they place on sports participation (50).

Alongside traditional gender roles where women are expected to take up the bulk of domestic and caretaking labour, sports participation often carries a higher opportunity cost, resulting in it falling lower on the list of prioritisations amid competing family and work commitments (2).

#### 1.6.3 Organisational and structural factors

From a pragmatic perspective, tangible barriers such as access to facilities and cost can limit or deter actual sports participation despite psychological readiness and motivation. Sports engagement requires access to suitable infrastructure such as courts, parks or fields to prevent injury and accidents. However, inequitable access may arise from imbalanced distribution of facilities between locations such as urban and rural areas or lack of maintenance in existing infrastructures (3, 4), creating environments perceived as unsafe. Though these are universal problems, women tended to show stronger avoidance effects as safety concerns outweigh physical activity, and thus are more likely to opt for sedentary activities instead (5). From a financial standpoint, women encounter systemic inequities within sports organisations, including limited funding, sponsorship, equipment, and international exposure, while female athletes also face the gender pay gap (51–54), aggravating the economic cost of sports participation, especially as a professional career. In addition, the field of sports medicine and by extension, injury prevention knowledge does not fully account for women in sports on the account of biological sexual dimorphism, with an overwhelming proportion of research only including male data (53, 55). This demonstrates the longstanding struggle of legitimising women's sports within a system deeply rooted in masculine dominance (52).

The undervaluation of women's sports extends into sports media. Female athletes tend to be under-reported and sidelined in media coverage, be it news reporting, photographs or airtime on televised programmes, a problem observed across multiple countries (36, 56, 57). Though one might assume the media landscape reflects audience interest, researchers found most agencies and editors do not base these decisions on formal data (58, 59). The discrepancy stemming from biased assumptions of the male dominated field of sports media again feeds back into the cycle of unfavourable attitude and lack of attention towards women in sports.

### 1.7 Women in sports-related leadership: a compounded gender gap

Women in sports leadership facilitates advocacy for more inclusive policies by representing women's perspective and needs, as well as uplifting women in sports as a mentor and role model outside of the male-centric networking, and as such are key players in transforming the existing sports landscape (60). From an organisational standpoint, diversity is simply a matter of business sense. Leadership studies demonstrated diversity policies opens access to a broader talent pool and reduces potential underutilisation of the workforce, bringing forth improved effectiveness, performance and stakeholder representation (61, 62).

Regardless, the gender inequality gap is typically even more pronounced at the sports leadership level. For instance, though the 2024 Paris Olympics achieved a landmark milestone of full gender equality (88), this representation failed to be reflected at the decision-making level, where women consisted of only 33% in the director level and 43% International Olympic Committee membership (63, 88). Historically, the pipeline theory, which posits observed inequality as a reflection of the lack of qualified women in the talent pool (64), is widely accepted. However, modern literature has shown its insufficiency to fully address the issue, seeing the discrepancy persisted despite women's increasing sports participation. Specifically, it fails to acknowledge systemic barriers embedded within the male dominant sports leadership structure that reproduces leadership as a male prerogative (6), which go by largely undetected due to their subtle and taken-for-granted nature. These may include a culture that embeds stereotypes of women in leadership, normalising destructive biases such as women are less qualified and committed to their roles, ultimately contributing to a non-inclusive environment where women are subjected to reduced access to over-scrutiny, alienation and wage inequalities (8–10).

The literature shows that gender inequality in sports is shaped by interconnected socio-cultural, internal and organisational factors which compounded effects for women in leadership positions (2, 8, 10, 35, 36, 40, 41, 47, 48, 51–54, 56–59, 62). However, research within the Malaysian context remains limited. While statistical gaps in participation and leadership are documented, there is insufficient understanding of the psychological resources that may empower Malaysian women to navigate this challenging landscape. Furthermore, the lived experiences that contextualise these quantitative trends, particularly how women interpret and cope with barriers, are not well captured by existing data.

Taken together, women's participation and sustained engagement in sports can be understood as a dynamic psychosocial system, where self-efficacy drives participation and persistence, emotions (positive or negative affect) influence motivation and engagement, and problem-solving enables coping with challenges. All these factors together can account for building resilience which helps to sustain continuous involvement in sports despite barriers. In a broader context, resilience is also dynamically shaped by external structures, including social-cultural norms, and institutional opportunities and constraints (65). Therefore, by understanding these psychological attributes (e.g., internal sources) and integrating the broader (external) socio-cultural and institutional considerations, more targeted supports can be provided to women in developing the resilience needed to overcome barriers and challenges (66), and succeed in sports engagement and leadership.

To address these gaps, this study employed a mixed-methods approach to explore the experiences of women in sports participation and sports-related leadership roles in Malaysia. In particular, this study investigated women resilience in sports in terms of self-perceived efficacy, emotions, self-rated problem-solving, self-assessed leadership and barriers to continuous sports participation. An online survey was employed to examine how leadership and teamwork self-efficacy, positive and negative emotions, and problem-solving skills accounting for resilience and sports' participation among Malaysian women, while the focus-group interviews were conducted to explore the personal and sociocultural narrative behind these statistical relationships. By integrating these data, this study hoped to provide a nuanced evidence base to inform strategies for supporting Malaysian women in sports.

## 2 Methods

### 2.1 Study design

This study used a convergent mixed-methods design. Quantitative (cross-sectional online survey) and qualitative (focus group) data were collected and analysed separately but concurrently. All materials were available in English and Malay language based on participant's preference to ensure the widest range of accessibility. Participants received RM20 for completing the online survey and RM50 for participating in the focus group. Ethical approval was granted by both National Sports Institute of Malaysia (RE/A/008/2025-016/2025) and University of Nottingham Malaysia (CPL150825).

### 2.2 Participant recruitment

Participants were Malaysian women aged 18 and above, recruited via convenience sampling through the distribution networks of the University of Nottingham Malaysia and the National Sports Institute, primarily through e-mail and WhatsApp. All participants provided written informed consent before taking part, and their participation was voluntary. No identifying information was shared with third parties.

### 2.3 Quantitative survey

#### 2.3.1 Participants and procedure

A target sample size was determined using G*Power 3.1 power analysis (67) based on a power of .95 and medium effect size of 0.30 to detect a two-tailed correlation, which suggested a target participant number of 138. The number was increased by 15%–25% to account for potential data exclusion rates typically observed in online surveys (68), which was then rounded up to the nearest hundred.

A total of 262 responses were collected from the online survey. Seventy-one were removed due to incomplete responses (*n* = 56) or because answers were based on physical activity instead of sports (*n* = 15). The final sample included in the data analysis consists of 191 participants, aged 18–63 years (*M* = 28.98, SD = 7.20).

#### 2.3.2 Materials

All survey materials were administered via Qualtrics and took approximately 45 min to complete. The survey consisted of (1) Demographic section, (2) Leadership/Teamwork Self-Efficacy Scale (LSES), (3) The Resilience Scale (RS), (4) Positive and Negative Affect Schedule (PANAS), (5) Shortened Problem-Solving Skills (PSI-20), and (6) Reflection scale on leadership self-assessment. Respondents were instructed to respond to these scales in relation to their experiences in sports. Demographic questions regarding factors that influence sports participation were included to determine participant's age, employment status, access to sports facilities (see Supplementary Appendix A). A complete methodology based on CHERRIES checklist guidelines are provided in Supplementary Appendix B.

##### 2.3.2.1 The resilience scale (RS)

This scale comprised 25 self-rating items, using a Likert-point of 1 (strongly disagree) to 7 (strongly agree). Sample items include “When I make plans I follow through with them”, “I usually manage one way or another”, “I am able to depend on myself more than anyone else”. Responses are summed to produce a total score, with a higher score indicates higher resilience. This scale has high reliability (internal consistency *α* = .91), test-retest reliability (*r* = .67–.84). This scale also has Cronbach's alpha coefficients ranging from .72 to .94 across 12 studies, supporting the internal consistency (15).

##### 2.3.2.2 Leadership/teamwork self-efficacy scale (LTSES)

This scale comprised 10 self-rating items, on a 5-point Likert-type from 1 (strongly disagree) to 5 (strongly agree), measuring individual's confidence in their leadership and teamwork abilities. Sample items include “I can energise my followers to achieve his/her best”, “I can determine what leadership style is needed to each situation”. Items 1, 2, 3, 5, and 10 contribute to teamwork factors, whilst items 4, 6, 7, 8, and 9 contribute to leadership factors. Responses for each type of factor were averaged across the number of items. A higher score in each factor indicates a higher self-efficacy in teamwork and leadership, respectively. The scale has established a high internal consistency of *α* = .90 (21).

##### 2.3.2.3 Positive and negative affect schedule (PANAS)

This scale consisted of a total 20 items (10 for PA and 10 for NA). Each item is a word that describes a specific feeling or emotion (e.g., interested, strong, enthusiastic, for PA; afraid, nervous, jittery, for NA). Respondents rated the extent to which they have experienced each emotion in a Likert scale, ranging from 1 (very slightly or not at all) to 5 (extremely). The scores were calculated by summing the rating for the respective items. Higher scores on the PA and NA scales indicate higher levels of PA and NA, respectively. This scale has been shown to have high internal consistency, with Cronbach's alpha coefficients typically above .80. It also demonstrates good convergent and discriminant validity, making it a reliable and valid measure of affect (26).

##### 2.3.2.4 Shortened problem-solving skills (PSI-20)

The PSI-20 is a 20-item short form of the Problem-Solving Inventory (69) that retains the three dimensions of problem-solving: confidence, approach-avoidance style, and personal control. It is designed to provide a viable alternative for assessing problem-solving skills with fewer items. A sample, for problem-solving confidence include “I am usually able to think up creative and effective alternatives to solve a problem”, for approach-avoidance style include “When faced with a difficult problem, I tend to avoid dealing with it”, and for personal control include “I feel in control of the problems that arise in my life”. This scale has a high internal consistency, with Cronbach's alpha coefficients >.70 and item reliability of >.80 for all three dimensions, respectively. Each item is rated on a 5-point Likert scale, ranging from 1 (Strongly agree) to 6 (Strongly disagree). Approach-avoidance questions were reverse coded. The scores were calculated by summing the responses to items in each dimension. Higher scores indicate better problem-solving abilities in the respective dimensions.

##### 2.3.2.5 Leadership self-assessment (LSA)

This is a comprehensive set of questionnaires that covers eight components, dealing with providing direction, leading courageously, fostering teamwork, championing change, coaching people, motivating others, building relationships, and acting with integrity (70). Each component has a set of five statements reflecting various attributes and skills. Respondents were asked to choose from three choices: 1 (I do not possess this attribute or do this skill well at all), 2 (I seldom possess this attribute or do this skill somewhat well), or 3 (I possess this attribute or do this skill very well). The scores were calculated by adding all responses in each component and dividing by 5. Each score indicates average scores for each leadership component (70).

### 2.4 Focus group interviews

Two focus group interviews were conducted separately: (1) six women who are active in sports (aged 19–29), (2) seven women who are not active or have withdrawn from sports participation (aged 30–47). A fuller demographic table and other relevant details are provided in Supplementary Appendix C.

Each session lasted approximately 60 min and was conducted in a respectful and informal setting to encourage open discussion. Two moderators who were fluent in English and Malay language facilitated the sessions using a semi-structured interview protocol, which ensured consistency across groups while allowing participants to elaborate on their experiences. The interview questions focused on four major domains: (1) Personal factors—motivation, self-efficacy, and emotional benefits of sports, (2) Sociocultural influences—family support, gender norms, and societal attitudes and cultural barriers, (3) Economic and structural barriers—financial considerations, accessibility of facilities, and funding opportunities, (4) Policy awareness—knowledge of gender equality initiatives and sports development programmes. Representative questions are provided in Supplementary Appendix D.

### 2.5 Data analysis

#### 2.5.1 Survey data analysis

Data from the online survey were analysed using SPSS software (Version 31.0.0.0). Descriptive statistics were tabulated to describe the characteristics of the respondent sample.

Several inferential analyses were conducted: (1) Spearman correlation to analyse relationships between PA, NA, LTSE, PSI and resilience, (2) Multiple linear regression to analyse the accountability value of PA, NA, LTSE, PSI on resilience, and (3) Mann–Whitney *U* to compare LSA between Leaders and Non-Leaders. A significance level of *p* < .05 was used for all statistical tests.

#### 2.5.2 Focus group data analysis

All discussions were audio-recorded with participants' informed consent. The sessions were conducted in English and Malay (i.e., participants were free to respond in either language they were more comfortable with), with occasional mixing of Chinese. When a participant used Malay or Chinese phrases, the moderators (both trilingual Malaysians) provided real-time clarification: one led the semi-structured questioning, while the other posed follow-up probes to ensure shared understanding and accurate audio capture. All discussions were transcribed verbatim, and the data were anonymised to protect confidentiality.

The lead moderator (i.e., second author) translated all Malay and Chinese-language responses into English. To ensure accuracy, the second moderator (i.e., first author) reviewed the translations for consistency, with particular attention to culturally specific terms. During analysis, the coder referenced the original transcripts whenever interpretive ambiguity arose.

Transcribed data were analysed using thematic analysis following Braun and Clarke's (71) six-phase framework of familiarisation, coding, theme identification, reviewing, defining, and reporting. This approach facilitated systematic comparison of codes across the Active and Inactive groups. The lead moderator transcribed the coded transcript manually, using printed copies with highlighters and marginal notes. Manual coding was chosen over software due to the multilingual data, which required close attention to original language nuances alongside English translations. A hybrid inductive-deductive approach was adopted: the four interview domains provided a loose deductive structure, but theme identification was predominantly inductive.

As coding was conducted by a single researcher, inter-coder reliability was not calculated. To enhance trustworthiness, (a) the lead moderator engaged in reflexive memo-writing throughout the coding process to document interpretive decisions, and (b) preliminary themes were reviewed by another moderator (who did not co-code but checked for consistency and clarity). A reflexive statement, as suggested by Rind (72), is provided in Supplementary Appendix E.

## 3 Results

Full demographic details of survey respondents are present in Supplementary Appendix F.

### 3.1 Correlations of emotional, cognitive and behavioural domains with resilience

Spearman's rank correlation was computed to assess the relationships between LTSES, PANAS (PA and NA) and PSI scores with resilience. LTSES, PA, and PSI were all positively and significantly correlated with resilience, while NA was found to have a significant inverse relationship with resilience (see Table 1).

**Table 1:** Correlation matrix table showing the relationships between PA, NA, LTSES, PSI and resilience*.*

Variable	PA	NA	LTSES	PSI
PA	–			
NA	−.18*	–		
LTSES	.56**	−.13	–	
PSI	.42**	−.36**	.44**	–
Resilience	.76**	−.34**	.63**	.56**

* *p* < .05.

** *p* < .001.

### 3.2 Variance explained by emotional, cognitive, and behavioural domains in resilience

Linear regression was used to test four independent factors (LTSE, PA, NA and PSI) to account for resilience scores (see Table 2).

**Table 2:** Summary of coefficients of regression.

Variable	Resilience		
	*B*	*β*	*p*
LTSES	12.17	.35	.00
PA	.92	.43	.00
NA	−.33	−.15	.00
PSI	.27	.17	.00

A significant regression was found *F*_(4,186)_ = 119.70, *p* ≤ .00). The *R*^2^ value was .72, indicating that LTSE, PA, NA and PSI explained approximately 72% of the variance in resilience. The regression equation was:

Resilience=39.27+(12.17xLTSES)+(.92xPA)+(−.33xNA)+(.27xPSI)

### 3.3 Estimation capacity of resilience with sports participation

Direct logistic regression was performed to assess the impact of resilience score on the likelihood that women would report that they were active in sports. The model contained 1 independent variable (RS score). The model was statistically significant, *χ*^2^ (1, *n* = 191) = 13.63, *p* < .001, indicating that it was able to distinguish between respondents who reported to be active and non-active in sports, respectively. The model as a whole explained between 6.9% (Cos and Snell *R* square) and 9.6% (Nagelkerke *R* squared) of the variance in sports participation status, and correctly classified 70.2% of the cases.

Resilience was a statistically significant factor to sports participation status, recording an odds ratio of 1.04, indicating that participants who reported higher resilience scores were 1.04 times more likely to report that they were active in sports.

### 3.4 Leadership self-assessment

The LSA tabulation (as shown in Table 3) showed that women in the current study had an overall moderate assessment of their leadership skills in the eight components. Respondent demographic details between leaders and non-leaders can be found in Supplementary Appendix G. The internal consistency of LSA questionnaire subscales was also conducted (see Supplementary Appendix H.

**Table 3:** Summary of median LSA subscales scores by leader and non-leader groups.

Leadership components	Leader		Non-leader	
	*n*	Md	*n*	Md
Providing direction	53	2.40	138	2.60
Leading courageously	53	2.40	138	2.40
Fostering teamwork	53	2.40	138	2.40
Championing change	53	2.60	138	2.40
Coaching people	53	2.40	138	2.40
Motivating others	53	2.60	138	2.40
Building relationships	53	2.40	138	2.40
Acting with integrity	53	2.80	138	2.80

A Mann–Whitney *U* Test revealed no significant differences in the LSA across subscale scores of Leaders and Non-Leaders, *U* = 3,662.50, *z* = .02, *p* = .99, *r* = .00, suggesting comparable self-assessment when asked to reflect on their leadership skills regardless of whether they are actually holding a leadership position.

### 3.5 Focus group interviews

Focus group interviews revealed key themes shaping their participation and leadership experiences. When asked to describe sports in one word, the Active group used terms like “passion”, “fun”, “energy” and “commitment”, reflecting their current engagement, youthful drive, and intrinsic motivation toward sports. Conversely, the Inactive group emphasised relational and wellbeing-oriented meanings rooted in past experiences, using words like “networking,” “making friends,” “health,” and “discipline.” Despite their differences, both groups associated sports with “competitiveness” and “achievement”, suggesting that engagement in sport, current or past, remains tied to a desire for challenge and accomplishment. Thematic analysis of the focus group interviews identified five key themes reflecting how structural, sociocultural, motivational, digital and institutional factors intersect to shape Malaysia women's participation and leadership in sports. Illustrative quotes from participants supporting each theme are provided in Supplementary Appendix I.

#### 3.5.1 Theme 1: barriers to sustained sports participation

Both Active and Inactive participants described substantial barriers that restricted their continued engagement in sports. The most pervasive challenge was competing responsibilities and role conflict, with commitments to work, studies, and family duties consuming the time and energy required for regular sports. Closely linked were financial costs related to transport, training, and coaching expenses, whilst equipment and facility fees also shaped the extent of their sporting involvement.

Environmental and logistical limitations further associated with disengagement. Participants highlighted the inaccessibility or poor maintenance of facilities, requiring significant travel to safe and suitable venues. “I used to stay opposite of Bukit Jalil, so I could just come down to run or swim. But now, there's no facilities nearby… and the parks are not well kept.” (Inactive Participant)

Embedded within this was a salient concern for personal safety, particularly when exercising alone. “If I want to go to a proper garden, I have to travel 20 min… running alone feels unsafe.” (Inactive Participant)

Participants also mentioned health-related disruptions, including injuries, weight changes, and the impact of the COVID-19 pandemic, were also cited as reasons for reduced participation or complete withdrawal from competitive involvement.

#### 3.5.2 Theme 2: gender influence and societal expectations

Sociocultural norms surrounding gender powerfully influenced participants' experiences, particularly in contexts traditionally perceived as male dominated. Participants described receiving biased perceptions of their physical capability and criticism of their appearance when participating in sports perceived as masculine or strength oriented. “People said I’m getting too big… are you even a girl?” (Active Participant) “If you’re doing a sport that needs you to gain muscles and tan, then automatically they try to discourage you.” (Active Participant) “When men fight, they look very man. When women fight… they say I look macam lelaki sikit la (a bit like a man)…My guy friends said, it's because you do martial arts… you don't get a boyfriend because you’re too masculine.” (Inactive Participant)

These comments reflected a deep-seated cultural preference for femininity associated with beauty standards emphasising thinness and skin fairness, creating a conflict between athletic and gendered identities. This reflects processes of gender performativity, identity negotiation, and stereotypes threat experienced by women athletes as they navigate the sociocultural constructed expectations (46, 73, 74), placing them under pressure when participating in sports that are socially defined as gender-incongruent. Despite these challenges, many women demonstrated strong behavioural resilience and empowerment. Participants also actively resisted gendered expectations by improving their performance, pursuing leadership roles, and creating opportunities for other female athletes to gain recognition. Through coaching, mentoring, and team leadership, they asserted their competence and challenged entrenched stereotypes.

#### 3.5.3 Theme 3: community, motivation, and leadership

Social and motivational factors were key drivers of the sustained involvement for the participants. Community, belonging, and social support emerged as foundational, with participants emphasising the importance of teammates, mentors, and family encouragement for offering emotional and logistical support especially during early involvement or transitions between levels of competition. “For me, it was community… people who have been in sports for a long time are willing to teach you and guide you.” (Active Participant)

This support system provided both practical guidance and emotional reinforcement. Intrinsic motivations such as passion, enjoyment and personal growth were strong drivers of continued engagement. Participants also reflected on their longstanding commitments to their sports, including representation at state-level competitions and early positive exposure during childhood. Even among those who became inactive due to life-stage changes, family responsibilities or mental health challenges, many remained connected to sports through coaching, administrative roles or recreational involvement. “I chose to work in sports because I feel comfortable in this environment… I’ve always wanted to stay in the sports world.” (Inactive Participant)

#### 3.5.4 Theme 4: the role of social media in empowerment

Digital platforms were identified as a significant positive influence. Participants used online platforms as sources of inspiration, representation and social connection that broadened their understanding of what sports can look like for women. “Social media helps more people to see women in sports…it normalises being strong and confident.” (Active Participant) “I saw one lady, 80 years old, who ran a marathon… that gave me inspiration that age is not crucial.” (Inactive Participant)

Exposure to athletes of different ages, body types, and abilities served to normalise strength and athleticism for women, challenged narrow beauty standards, and provided relatable role models outside their immediate circles, thus complementing offline communities.

#### 3.5.5 Theme 5: policy awareness and institutional support

A clear gap existed between participants' experiences and formal sports structures. Participants from both groups expressed limited awareness of existing gender-equity policies, athlete development programmes or institutional mechanisms aimed at promoting women's participation in sports. Many admitted uncertainty about available resources, coaching pathways, or national-level initiatives, reflecting a disconnect between grassroots involvement and existing policy structures.

**Figure 1:**
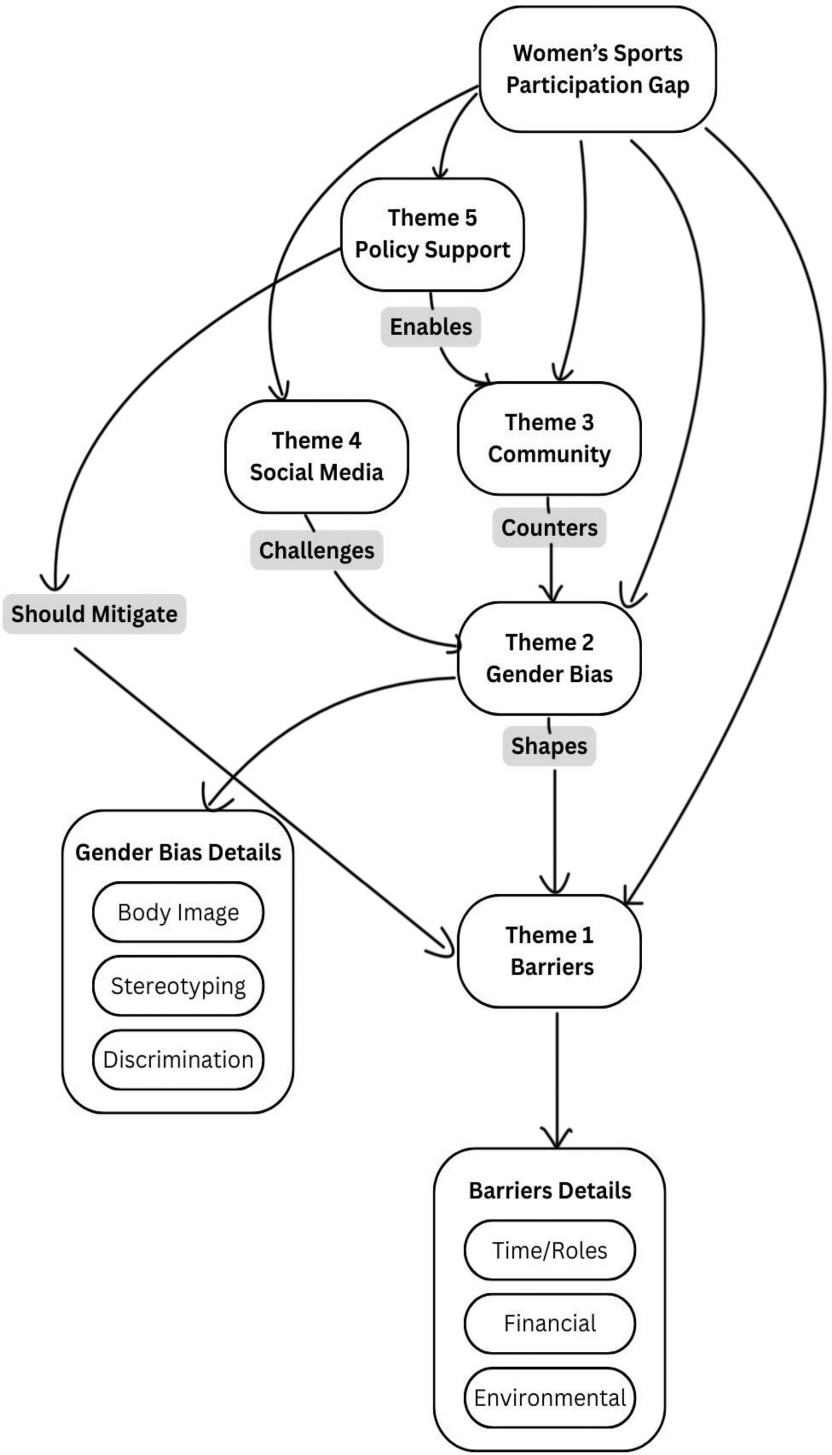
Conceptual model of thematic interactions in women's sports participation. Barriers to participation (Theme 1) are positioned as a foundation and are shaped by gender bias (Theme 2). Community influences (Theme 3) and social media (Theme 4) may counteract these challenges, while policy support (Theme 5) functions as an enabling factor in mitigating barriers. Subthemes for barriers (time/role constraints, financial, and environmental factors) and gender bias (e.g., body image concerns, stereotyping, and discrimination) are indicated. Arrows represent proposed directional relationships between the five primary themes based on participants’ reported experiences.

Barriers such as high costs for official accreditation and lack of promotion for non-mainstream sports were noted. However, some participants acknowledged recent positive developments attributed to the current Minister of Youth and Sports, Hannah Yeoh. Increased governmental involvement, recognition of previously underfunded sports, and expanded opportunities for women's competitions were perceived as positive shifts. Figure 1 illustrates how these themes operate in relation to one another.

## 4 Discussion

This study presents a nuanced understanding of the Malaysian sports landscape navigated by women. By assessing how different factors account for variance in resilience building among women alongside qualitative narratives of women's lived experiences in sports, the findings present evidence towards the link of emotional, cognitive and behavioural domains to women's resilience, as well as revealing persistent structural and sociocultural barriers. As illustrated in Figure 1, women also identified the enabling factors of community, digital media and inclusive leadership in supporting their sports participation.

### 4.1 Relationships between LTSE, PA and PSI with resilience

Quantitative analysis established a robust model for resilience among Malaysian women in sports. Significant positive correlations were found between resilience and leadership/teamwork self-efficacy (LTSE), positive affect (PA), and problem-solving skills (PSI), with a significant negative correlation for negative affect (NA) with resilience. This supported the concept of resilience as a multifaceted construct of which involved cognitive, emotional and behavioural domains. This finding corroborated of Pillay-Naidoo and Nel (18), who similarly reported empirical support of the accountability of these factors, as well as extended the work by providing cross-cultural support via its relevancy within the Malaysian population. Further regression analysis indicated all factors were significant account for variance in resilience, with PA being the most prominent factor, followed by LTSE, PSI, then NA. The most prominent role of PA in accounting for resilience in the current study is not dissimilar from previous findings where cognitive or behavioural assets, when examined alongside PA accounted for stronger correlations towards resilience compared to when examined in isolation (18). Although resilience has been found as a significant yet relatively limited role in explaining women's participations in sports, this may be attributed to external factors—such as limited access to sports facilities and economic constraints—that hinder actual participation despite strong intentions.

These findings offer support towards prioritising PA in future resilience-building research or interventions, especially emotions indicating motivation and interest per PA items in PANAS. Focus group responses suggest supportive sports communities, female role models on social media, and inclusive leadership as important avenues for promoting PA among women in sports.

### 4.2 Women's self assessed leadership skills

Overall, women had moderate scores on their self-assessed leadership skills. Based on the data gathered, women's current leadership strength lied within their ability to conduct according to their values (e.g., acting with integrity), while areas requiring further development were mainly interpersonal (e.g., leading courageously, fostering teamwork, coaching people, building relationships). This is consistent with trends where female-led organisations tended to conduct more ethically (75, 76), as well as the reality faced by female leaders, where associations of leadership as a masculine trait remain prevalent (77), which can result in lower self-rated leadership abilities due to perception of own-gender incongruence. These findings potentially suggest that women may internalise sociocultural constructed norms about leadership expectations, limiting the perceived legitimacy of alternative leadership styles. This put them in a paradoxical situation—despite demonstrating resilient and agentic behaviours in practice, their traditionally shaped belief on gendered roles creates a persistent perception-reality gap, as demonstrated in a recent empirical study (78).

### 4.3 Interpreting barriers: structural stagnation and gendered negotiation

The qualitative findings reveal that barriers to women's sustained sports participation are not isolated but are deeply interconnected. The persistence of structural and logistical obstacles particularly concerns safety and facility access. This aligns with earlier Malaysian studies (3), suggesting limited progress in creating an inclusive physical infrastructure. The need to travel to “proper” and safe venues acts as a “participation tax” of extra time, cost, and logistical planning, which disproportionately burdens women.

This structural barrier is compounded by life-stage transitions, where the shift from the structured environment of university to the demands of career and family life systematically redefines sport as a dispensable luxury rather than an integral part of daily routine. This intersection creates a powerful funnel effect, where time poverty and logistical burdens converge to push women out of regular participation.

Although not always the primary driver of dropout, the pervasive policing of female athletes for appearing “too big” or “masculine” reflects a deep-rooted cultural tension between the strength and physique fostered by athleticism and restrictive local femininity norms that idealise thinness and fairness. As some participants noted, this policing extends beyond aesthetics to social desirability, framing sports involvement as a threat to traditional heterosexual milestones. These experiences are not merely discouraging comments but constitute a form of identity conflict, forcing women to constantly negotiate between their athletic selves and societal expectations of femininity and prescribed social roles. These lived experiences of women participating in sports mirror and reinforce established findings in the literature on gender performativity, identity negotiation, and stereotype threat (46, 79, 80).

Collectively, our findings indicate that Malaysian women navigate a dual burden: external, structural exclusion from spaces and time, and internal, sociocultural exclusion from fully claiming an athletic identity without sanction. In a more global context, particularly across Asian and Global South (81–83), comparable trends have also been documented where women's sport participation is challenged by similar psychological and sociocultural barriers, structural and institutional constraints, alongside ongoing gaps between policy intentions and lived experiences. This intersection indicates that disengagement is rarely due to a loss of interest but is often represents a constrained outcome of cumulative pressure.

### 4.4 An integrated perspective of quantitative and qualitative findings

The strength of this mixed-methods design lies in its capacity to use qualitative narratives to explain and expand the quantitative patterns, delving into how lived experiences contextualise psychology resources. This integration reveals that the psychological resources measured in the survey do not operate in a vacuum but are actively shaped by the barriers and enablers women navigate.

First, the finding that PA was the strongest factor in explaining variance in resilience is given depth by the qualitative data. While the survey identified PA's statistical importance, the focus groups revealed its social origins. Participants consistently described how supportive peer communities and visibility of relatable role models on social media were direct sources of excitement, interest, and enthusiasm—the very emotions captured by the PANAS scale. This suggests that emotionally supportive sporting environments can serve as counterspaces, buffering women against exclusionary gender norms while fostering sustained psychological engagement with sports, as demonstrated in recent studies (84–86). Therefore, interventions aiming to bolster resilience by increasing PA should not focus solely on the individual, but on cultivating the social ecosystems that generate these positive emotions.

Second, the moderate scores on interpersonal leadership skills (e.g., building relationships) in the self-assessment find their explanation in the described socio-cultural environment. The qualitative data detailed a landscape of gendered criticism and identity conflict, where women feared social sanction for being perceived as too assertive or “masculine.” This societal friction helps to explain why women might report lower confidence in interpersonal leadership: these skills demand assertive networking and influence—behaviours they have learned carry greater social risks. Thus, the quantitative score is not merely a lack of skill but may reflect a strategic adaptation to a perceived non-inclusive environment.

Finally, the quantitative link between PSI and resilience is reframed by the overwhelming narrative of structural barriers. Participants described problems like unsafe facilities and a lack of time as systemic and often unsolvable at the individual level. This suggests that high PSI might be less about overcoming these specific barriers and more about navigating around them—finding alternative times, venues, or activities—in order to persist. Resilience, in this context, is not about eliminating barriers but about sustaining the cognitive and behavioural efforts required to negotiate them continuously.

In summary, this integrated analysis shows that psychological resources like PA, self-efficacy, and problem-solving are not just internal traits but are dynamic responses to an external environment. They are fuelled by community, constrained by gendered norms, and deployed against structural obstacles. Effective support for Malaysian women in sports must therefore target this person-environment interaction.

### 4.5 New pathways: digital counter-narratives and the policy awareness gap

Against this backdrop, the study identified critical pathways that point toward solutions. Social media emerged as a vital arena for curating visible counter-narratives. Exposure to diverse athletes (e.g., an 80-year-old marathoner) normalises strength and expands “possible selves” (87), directly challenging restrictive stereotypes and complementing offline communities. Some participants' exposure to empowering digital content inspired self-belief and challenged traditional notions of ageing and femininity in sports (represented by connection between Theme 4 and Theme 2 in Figure 1).

However, a significant chasm between institutional policy and grassroots awareness was evident. Participants demonstrated limited knowledge of existing support programmes, highlighting a critical implementation gap. This also indicates that introducing gender-equity initiatives alone may be insufficient to ensure women perceive sports as accessible and experience meaningful support in their participation. However, their positive recognition of recent ministerial efforts suggests that communicative and representative leadership can directly improve perceptions of support and feasibility. This presents a strategic opportunity for institutions to strengthen engagement by leveraging clear and consistent communication, increasing programme visibility, and improving local dissemination to effectively reach target audience (represented by connections between Theme 5, Theme 3, and Theme 1 in Figure 1).

### 4.6 Limitations and future research

The study utilised a cross-sectional design and hence is unable to track nor draw conclusions on how the Malaysian sporting environment and its associated barriers might change over time. In addition, though the cognitive, emotional and behavioural domains of resilience were demonstrated, their effectiveness as components in resilience intervention remains to be explored. Additionally, as a preliminary exploratory study, data from this research were from self-selected, voluntary participants. As such, women who were completely inactive in sports may not sign up due to perceiving they are unrelated to the research. Notably, the views of women who have never engaged in sports and those from more marginalised or rural backgrounds may be underrepresented, potentially overlooking the most absolute barriers to entry. As an extension, the generalisation of these findings requires further studies, particularly related to the robustness of the cognitive, emotional and behavioural contributions to resilience and the LSA as a wider tool for Malaysian women.

Future research could consider longitudinal design to track whether and how the Malaysian sports landscape might shift over time, which could inform further understanding of underlying factors pushing transformation of the Malaysian sporting landscape. Further intervention-centric studies could examine the effectiveness of introducing alternative leadership framework, policy distribution, resilience/self-assessment informed methods in introducing changes and supporting women in sports.

## Data Availability

The raw data supporting the conclusions of this article will be made available by the authors, without undue reservation.
