# Supplementary material for: Exploring resilience in sports participation and self-assessed leadership among women in Malaysia: a mixed-methods approach
Source: Front Sports Act Living. 2026 Jul 6;8:1890468. doi: 10.3389/fspor.2026.1890468 (PMC13381797; doi:10.3389/fspor.2026.1890468)
Supplement: Supplementary file 1 [file Datasheet1.docx]

| **Question Number** | **Content** |
| --- | --- |
| 1 | What is your sex? |
| 2 | What is your current age? |
| 3 | What is your nationality? |
| 4 | In which state of Malaysia do you reside in? |
| 5 | What is your ethnicity? |
| 6 | What is your religion? |
| 7 | What is your highest qualification? |
| 8 | What is your marital status? |
| 9 | How many children do you have? |
| 10 | Which of the below would best describe your household income level? |
| 11 | What best describes your current employment status? |
| 12 | What sector is your occupation in? |
| 13 | Which of these best describe your job position? |
| 14 | In general, how would you describe your health? |
| 15 | Are you currently active in sports? |
| 16 | Are you involved in any sports leadership role? |
| 17 | Which of the below sports facilities do you have access to? |
| 18 | What type of sports support system do you have access to? |
| 19 | How would you best describe your level of sports participation? |
| 20 | What type of sports do you participate in? |
| 21 | On average, how many days in a week do you participate in sports? |
| 22 | How many years have you actively participated in sports? (Includes past participation) |

**Appendix A.** Demographic questions in survey.

Appendix B. CHERRIES checklist guidelines.

Four checkpoints were included as questions prior to the start of the survey body to ensure respondents qualify the participation criteria of Malaysian women who were 18 years and above. Participants who do not meet the participation requirements were directed to a rejection statement and were not able to proceed to the survey body.

All questions were mandatory, and only data from full survey completion were collected. Occupation related and sports participation related portions of the demographic section were adaptive, as these were only relevant to respondents who are currently working, and participants who have sports participation experience, respectively. Number of items per page averaged at 20, distributed over 7-8 pages (exact page number depends on adaptive questioning based on responses). Survey respondents were provided with a back button to navigate the survey. Multiple entries were prevented using the Duplicate Detection function on Qualtrics.

The surveys were password protected to comply with copyright holder terms, and were tested for usability and functionality by the research team prior to launching. A duration of approximately 1 month (5 October 2025 - 8 November 2025) was used to collect survey responses.

## **Appendix C.** Full demographic table for focus group interviews.

| Name | Group | Age | Ethnicity | Location | Sport(s) |
| --- | --- | --- | --- | --- | --- |
| P1 | Inactive | 35 | Chinese | Petaling Jaya, Selangor | Tang Soo Do, Netball, Basketball |
| P2 | Inactive | 45 | Chinese | Puchong, Selangor | Jogging, Swimming, Hiking |
| P3 | Inactive | 31 | Chinese | Kajang, Selangor | Ultimate Frisbee |
| P4 | Inactive | 37 | Malay | Seri Kembangan, Selangor | Squash |
| P5 | Inactive | 43 | Malay | Nilai, Negeri Sembilan | Badminton |
| P6 | Inactive | 48 | Malay | (not stated) | Handball, Netball |
| P7 | Inactive | 44 | Malay | Bukit Jalil, KL | Badminton |
| P8 | Active | 20 | Chinese | Kuantan, Pahang | Ultimate Frisbee |
| P9 | Active | 24 | Chinese | Puchong, Selangor | Volleyball |
| P10 | Active | 30 | Chinese | Petaling Jaya, Selangor | Track & Field, Cheerleading |
| P11 | Active | 24 | Indian | Cheras, KL | Badminton, Tennis, Golf, Pickleball |
| P12 | Active | 21 | Indian | Cheras, KL | Badminton |
| P13 | Active | 24 | Indian | Duta Kiara, KL | Ultimate Frisbee |

All participants resided in urban or peri‑urban areas of Peninsular Malaysia, specifically in Selangor, Kuala Lumpur, and Kuantan (Pahang). Based on name and location, ethnic backgrounds included Chinese (n = 6), Malay (n = 4), and Indian (n = 3). Sports engaged in spanned team disciplines (netball, Ultimate Frisbee, volleyball, handball, basketball) and individual or mixed disciplines (badminton, squash, jogging, swimming, hiking, Tang Soo Do, golf, track & field, competitive cheerleading). Among the inactive group, participation was historically recreational or school‑based; among the active group, participation ranged from regular recreational to competitive (e.g., competitive cheerleading, state‑level track & field).

Limitations of demographic data: Information on education level, marital/family status, household socioeconomic background, and formal leadership experience in sports was not collected during the focus group discussions. This missing information is particularly relevant for the inactive group (aged 30–47), where life‑stage factors such as marriage, motherhood, or career establishment may influence sports participation independently of inactivity status. We therefore interpret age‑related patterns with caution.

## **Appendix D.** Focus Group Interview Questions.

**Section 1: Personal & Internal Factors**

**For Active Participants:**

- One word that describes how you feel about sports today.
- What first inspired or motivated you to get involved in sports or take on a leadership role?
- What are some personal benefits or changes you’ve noticed in yourself because of your involvement in sports?
- Did you feel like the people around you supported your decision to get involved — or was it something you had to push for yourself?
- Have there been times when personal commitments or challenges made it hard to Continue?
- Optional follow-up: “When things became challenging, what helped you keep going?”

**For Inactive/Withdraw Participants:**

- One word that describes how you feel about sports today.
- What first inspired or motivated you to get involved in sports or take on a leadership role?
- While you were active, what personal benefits or changes did you experience?
- Did you feel like the people around you supported your decision to get involved — or was it something you had to push for yourself?
- What personal challenges or doubts led to your decision to step back from sports?
- Looking back, how do you feel about your decision to step away from sports — is it something you feel at peace with, or do you ever think about returning?
- *Optional follow-up*: “Is there anything that could have helped you stay involved longer?”

**Section 2: Socio-Cultural Influences**

**For Both:**

- Have you ever felt that your involvement in sports was questioned or misunderstood or judged by others?
- How have expectations around your role at home, work, or within your community affected your ability to stay involved?
- Do you feel that your gender influenced how others viewed or treated your participation in sports?
- Have cultural or religious expectations ever made it harder for you to stay involved in sports?
- *Optional follow-up*: Did your family or workplace ever challenge or question your participation?

**Section 3: Economic & Structural Barriers**

**For Active Participants:**

- Have financial or logistical barriers (like equipment costs, transport, time) made it difficult to stay involved?
- Do you feel there are enough facilities, funding, or opportunities available for women in your sport or community?
- Have you received any financial support or sponsorship for your involvement in sports?
- If so, what kind of financial or external support have you received, and how has that impacted your journey?
- How do you currently manage or overcome these financial/logistical challenges?

**For Inactive/Withdraw Participants:**

- Have financial or logistical barriers (like equipment costs, transport, time) made it difficult to stay involved?
- Do you feel there are enough facilities, funding, or opportunities available for women in your sport or community?
- Have you received any financial support or sponsorship for your involvement in sports?
- If so, what kind of financial or external support have you received, and how has that impacted your journey?
- Did financial limitations, work commitments, or lack of sponsorship contribute to your decision to step away?

**Section 4: Awareness and Perceptions of Policies**

**For Both:**

- Are you familiar with any policies or programs that support women in leadership or sports in Malaysia?
- From your perspective, are these efforts visible or making an impact?
- What suggestions do you have for improving support or policies?

**Closing**

- What changes or improvements would you like to see in the future for women in sports and leadership roles in Malaysia?
- What advice would you give to young girls interested in sports leadership?

**Appendix E.** Reflexive Statement.

The second author (lead moderator, coder) is a female Malaysian psychology graduate student with three years of competitive ultimate frisbee experience at the university level, as well as prior involvement in track & field and volleyball. This background was acknowledged as a potential source of bias, and care was taken during interview design to avoid leading questions.

**Appendix F.** Demographic information of respondent sample.

|  | **n** | **%** |
| --- | --- | --- |
| **State** |  |  |
| Johor | 15 | 7.85 |
| Kedah | 9 | 4.71 |
| Kelantan | 3 | 1.57 |
| Kuala Lumpur | 39 | 20.42 |
| Malacca | 3 | 1.57 |
| Negeri Sembilan | 6 | 3.14 |
| Pahang | 8 | 4.19 |
| Penang | 11 | 5.76 |
| Perak | 12 | 6.28 |
| Perlis | 6 | 3.14 |
| Putrajaya | 9 | 4.71 |
| Sabah | 1 | 0.52 |
| Sarawak | 3 | 1.57 |
| Selangor | 61 | 31.94 |
| Terengganu | 5 | 2.62 |
| **Ethnicity** |  |  |
| Chinese | 86 | 45.03 |
| Indian | 10 | 5.24 |
| Malay | 94 | 49.21 |
| Punjabi | 1 | 0.52 |
| **Religion** |  |  |
| Buddhism | 61 | 31.94 |
| Christianity | 23 | 12.04 |
| Hinduism | 9 | 4.71 |
| Islam | 94 | 49.21 |
| No religion | 3 | 1.57 |
| Sikh | 1 | 0.52 |
| **Income level (Based on Household Income Survey Report 2022 (Malaysia & States [79])** |  |  |
| High income  (Urban ≥ RM 10,710; Rural ≥ RM 6,210) | 53 | 27.75 |
| Middle income  (Urban: RM 5,110-RM 10,709; Rural: RM 3,100-RM 6,209) | 88 | 46.07 |
| Low income  (Urban < RM 5,110; Rural < RM 3,100) | 50 | 26.18 |
| **Employment status** |  |  |
| Employed | 149 | 78.01 |
| Unemployed | 4 | 2.09 |
| Student/Retired/Home-maker | 38 | 19.9 |
| **Job level** |  |  |
| Manager and above | 35 | 23.49 |
| Individual contributor | 72 | 48.32 |
| Entry level | 42 | 28.19 |

**Appendix G.** Demographic breakdown by leadership and non-leadership status of respondent sample.

|  | **Leader** | **Non-Leader** |
| --- | --- | --- |
| **n** | 53 | 138 |
| **Mean Age** | 31.30 | 28.09 |
| **SD Age** | 8.65 | 6.37 |
| **Education Level** |  |  |
| High school graduate and below | 3 | 17 |
| Diploma/Vocational Degree | 13 | 36 |
| Undergraduate | 28 | 60 |
| Postgraduate | 9 | 25 |
| **Marital Status** |  |  |
| Unmarried | 31 | 45 |
| Married | 22 | 93 |
| **Employment Sector** |  |  |
| Private | 35 | 76 |
| Public | 6 | 27 |
| Non-profit | 3 | 2 |
| Not employed | - | 33 |

**Appendix H.** Reliability analysis of Leadership Self-Assessment (LSA) questionnaire subscales.

| **Subscale** | **n (number of items)** | **α** |
| --- | --- | --- |
| Providing direction | 5 | 0.82 |
| Leading courageously | 5 | 0.72 |
| Fostering teamwork | 5 | 0.82 |
| Championing change | 5 | 0.86 |
| Coaching people | 5 | 0.82 |
| Motivating others | 5 | 0.85 |
| Building relationships | 5 | 0.84 |
| Acting with integrity | 5 | 0.86 |

**Appendix I.** Participant Quotations Organised by Theme.

| **Theme** | **Subtheme** | **Illustrative Quotes** |
| --- | --- | --- |
| **Barriers to Sustained Sports Participation** | **Time constraints, role demands and life-stage transitions** | **“Because of school,I’m losing passion…it’s hard to commit…” (Active)**  **“I have two children and then I am in LDR. My husband works elsewhere…so I don’t have time for sports and activities because of my daily routine.” (Inactive)**  **“Once you start working, you can’t train every night like in uni. You just want to go home and rest.” (Active)** |
|  | **Financial and logistical challenges** | **“Sometimes I just don’t want to spend money to grab training.” (Active)**  **“Tournament fees are expensive… Transport can also be a challenge since some tournaments are far but we manage by carpooling.” (Active)**  **“Private coach costs RM180 per hour… international coach USD 250 per hour.” (Inactive)** |
|  | **Environmental and safety challenges** | **“If I want to go to a proper garden, I have to travel 20 minutes…running alone feels unsafe.” (Inactive)**  **“I used to stay opposite of Bukit Jalil, so I could just come down to run or swim. But now, there’s no facilities nearby… and the parks are not well kept.” (Inactive)** |
|  | **Injuries, health concerns, and COVID-related disruptions** | **“I played Frisbee for 10 years but I stopped once COVID happened. After 2 years, it's very hard to build back to play in a team…my knees are kind of hurting…because that time was like uni time so I think I played like five days a week, so the transition…suddenly COVID, not playing competitive sport at all, and to come back…it’s very effortful.” (Inactive)**  **“Bila kerja saya masih join netball… then I stop sport ialah bila saya dah injured…meniscus tear so tak pernah main langsung.” (Inactive)**  **“I also knee feel… knee pain…So I slow down. And then, after I got married. I ate so many and I gained 10kg.” (Inactive)** |
| **Gender Influence and Societal Expectations** | **Judgement about body, physique and femininity** | **“People said I’m getting too big…are you even a girl?” (Active)**  **“The boys were always like, oh all the girls are weak or they’re not strong enough.” (Active)**  **“The beauty standard now is that you have to be skinny, pale skinned and everything. So, let's say you are doing a sport that needs you to gain muscles and like tan, then automatically they try to discourage you from doing the sports.” (Active)** |
|  | **Gender stereotyping and discrimination in male-dominated sports** | **“My mom scolded me once for being tan after a tournament”. (Active)**  **“When men fight, they look very man. When women fight… they say I look macam lelaki sikit la. I was like, why the discrimination? My guy friends said, it’s because you do martial arts… you don’t get a boyfriend because you’re too masculine.” (Inactive)**  **“When it’s a male category competition…the crowd was full. When women fight, it’s quiet…People think martial arts are only for guys.” (Inactive)** |
|  | **Using discrimination as motivation** | **“In the beginning I used to feel like, okay what am I doing? Why am I not getting the disc?... then I try to work towards…show them what I’m capable of doing.” (Active)** |
|  | **leadership as resistance and empowerment** | **“If you’re not strong enough, it just feels invisible for them.” (Active)**  ***“I give female athletes leadership positions so they can prove themselves”*. (Active)** |
| **Community, Motivation, and Leadership** | **Importance of community, belonging and mentorship** | **“For me, it was community… people who have been in sports for a long time are willing to teach you and guide you.” (Active)**  **“Being president for badminton and ping pong was a way to show my juniors that this is a sport you can play.” (Active)** |
|  | **Social encouragement and family support** | **“I think what inspired me…at first is friends because I have been selected for …volleyball since my primary school, which is convinced by my friends.” (Active)**  **“All the sport equipment and stuff is from family.” (Active)** |
|  | **Intrinsic motivations and long-term engagement** | **“I constantly had to remind myself that I can do more and I can do more, maybe not in height but I can provide in different ways.” (Active)**  **“I started martial arts when I was nine… I represented Perak for two years.” (Inactive)**  **“Saya main handball and netball… I played in MSSM and represented Johor.” (Inactive)** |
|  | **Adaptive resilience despite life-stage changes** | **“I chose to work in sports because I feel comfortable in this environment… I’ve always wanted to stay in the sports world.” (Inactive)**  **“My mental health got bad… I saw a therapist before I retired.” (Inactive)** |
|  | **Need for supportive communities** | **“Maybe create a community where women can express themselves.” (Active)**  **“Maybe like a community where women can actually say how you feel? Without being judged… a free space for them to talk about everything.” (Active)** |
| **The Role of Social Media in Empowerment** | **Redefine perceptions of women in sport and challenge traditional norms of femininity and age.** | **“See a lot of women empowerment videos on Instagram…both platforms,” (Active)**  **“Social media helps more people to see women in sports…it normalizes being strong and confident.” (Active)**  **“Before that I would feel old already, but I saw one lady, 80 years old, who ran a marathon… that gave me inspiration that age is not crucial.” (Inactive)** |
| **Policy Awareness and Institutional Support** | **Low awareness of policies or pathways** | **“Are there any [policies]? I’m actually curious.” (Active)**  **“I don’t know about it… if I knew, maybe I would consider it.” (Inactive)** |
|  | **Barriers to certification and recognition** | **“To be a coach, you must take Sport Science Level 1, 2, 3… now it’s more strict.” (Inactive)**  **“Tang Soo Do wasn’t recognised… so no public funding, only private sponsors.” (Inactive)** |
|  | **Improved recognition under Hannah Yeoh (Minister of Youth and Sports)** | **“So fortunately, again, with social media. In recent years, Tang Soo Do is already recognized and Hannah Yoeh also already started funding…it's starting to get more recognition. But before that, no recognition. Hence, no public funding. So, it was private funding.” (Inactive)**  **“During my dance sports days, I did have support but they were private sectors…if you reach a certain level and you get recognized, then only sponsors will start to come in…Now with Hannah Yeoh’s support, the federation is under proper sports recognition and receives government support.” (Inactive)**  **“...they finally realised, we have to accept the fact that we can't go back to traditional ways. So now, they have more female competitions rather than you must be partner. In the past you must have a partner. If not, you can never compete.” (Inactive)** |

**Theme 1: Barriers to Sustained Sports Participation**

Time constraints, role demands and life-stage transitions

- “Because of school, I’m losing passion…it’s hard to commit…” (Active)
- “I have two children and then I am in LDR. My husband works elsewhere…so I don’t have time for sports and activities because of my daily routine.” (Inactive)
- “Once you start working, you can’t train every night like in uni. You just want to go home and rest.” (Active)

Financial and logistical challenges

- “Sometimes I just don’t want to spend money to grab training” (Active)
- “Tournament fees are expensive… Transport can also be a challenge since some tournaments are far but we manage by carpooling.” (Active)
- “Private coach costs RM180 per hour… international coach USD 250 per hour.” (Inactive)

Environmental and safety challenges

- “If I want to go to a proper garden, I have to travel 20 minutes…running alone feels unsafe.” (Inactive)
- “I used to stay opposite of Bukit Jalil, so I could just come down to run or swim. But now, there’s no facilities nearby… and the parks are not well kept.” (Inactive)

Injuries, health concerns, and COVID-related disruptions

- “I played Frisbee for 10 years but I stopped once COVID happened. After 2 years, it's very hard to build back to play in a team…my knees are kind of hurting…because that time was like uni time so I think I played like five days a week, so the transition…suddenly COVID, not playing competitive sport at all, and to come back…it’s very effortful.” (Inactive)
- “Bila kerja saya masih join netball… then I stop sport ialah bila saya dah injured…meniscus tear so tak pernah main langsung.” (Inactive)
- “ I also knee feel… knee pain…So I slow down. And then, after I got married. I ate so many and I gained 10kg... ”” (Inactive group)

**Theme 2: Gender Influence and Societal Expectations**

Judgement about body, physique and femininity

- “People said I’m getting too big…are you even a girl? ” (Active)
- “The boys were always like, oh all the girls are weak or they’re not strong enough.” (Active)
- “The beauty standard now is that you have to be skinny, pale skinned and everything.. So, let's say you are doing a sport that needs you to gain muscles and like tan, then automatically they try to discourage you from doing the sports.”(Active)

Gender stereotyping and discrimination in male-dominated sports

- “My mom scolded me once for being tan after a tournament”. (Active)
- “When men fight, they look very man. When women fight… they say I look macam lelaki sikit la. I was like, why the discrimination? My guy friends said, it’s because you do martial arts… you don’t get a boyfriend because you’re too masculine.” (Inactive)
- “When it’s a male category competition…the crowd was full. When women fight, it’s quiet…People think martial arts are only for guys.” (Inactive)

Using discrimination as motivation

- “In the beginning I used to feel like, okay what am I doing? Why am I not getting the disc?... then I try to work towards…show them what I’m capable of doing.” (Active)

leadership as resistance and empowerment

- “If you’re not strong enough, it just feels invisible for them.” (Active)
- *“I give female athletes leadership positions so they can prove themselves”*. (Active)

**Theme 3: Community, Motivation, and Leadership**

Importance of community, belonging and mentorship

- “For me, it was community… people who have been in sports for a long time are willing to teach you and guide you”. (Active)
- “Being president for badminton and ping pong was a way to show my juniors that this is a sport you can play”. (Active)

Social encouragement and family support

- “I think what inspired me…at first is friends because I have been selected for …volleyball since my primary school, which is convinced by my friends.” (Active)
- “All the sport equipment and stuff is from family” (Active)

Intrinsic motivations and long-term engagement

- “I constantly had to remind myself that I can do more and I can do more, maybe not in height but I can provide in different ways”. (Active)
- “I started martial arts when I was nine… I represented Perak for two years.” (Inactive)
- “Saya main handball and netball… I played in MSSM and represented Johor.” (Inactive)

Adaptive resilience despite life-stage changes

- “I chose to work in sports because I feel comfortable in this environment… I’ve always wanted to stay in the sports world.” (Inactive)
- “My mental health got bad… I saw a therapist before I retired.” (Inactive)

Need for supportive communities

- “Maybe create a community where women can express themselves.” (Active)
- “Maybe like a community where women can actually say how you feel? Without being judged… a free space for them to talk about everything.” (Active)

**Theme 4: The Role of Social Media in Empowerment**

Online platforms helped redefine perceptions of women in sport and challenge traditional norms of femininity and age.

- “See a lot of women empowerment videos on Instragram…both platforms,” (Active)
- “Social media helps more people to see women in sports…it normalizes being strong and confident.”(Active)
- “Before that I would feel old already, but I saw one lady, 80 years old, who ran a marathon… that gave me inspiration that age is not crucial.”(Inactive)

**Theme 5: Policy Awareness and Institutional Support**

Low awareness of policies or pathways

- “Are there any [policies]? I’m actually curious.” (Active)
- “I don’t know about it… if I knew, maybe I would consider it.” (Inactive)

Barriers to certification and recognition

- “To be a coach, you must take Sport Science Level 1, 2, 3… now it’s more strict.” (Inactive)
- “Tang Soo Do wasn’t recognised… so no public funding, only private sponsors.” (Inactive)
